# A protocol for preparation and transfection of rat entorhinal cortex organotypic cultures for electrophysiological whole-cell recordings

**DOI:** 10.1016/j.mex.2017.10.003

**Published:** 2017-10-18

**Authors:** Nicholas I. Cilz, James E. Porter, Saobo Lei

**Affiliations:** Department of Biomedical Sciences, School of Medicine and Health Sciences, University of North Dakota, Grand Forks, ND 58203, USA

**Keywords:** Biolistic transfection of entorhinal sections, Organotypic, Entorhinal, Biolistic, Slice, Transfection

## Abstract

Understanding how neuromodulators influence synaptic transmission and intrinsic excitability within the entorhinal cortex (EC) is critical to furthering our understanding of the molecular and cellular aspects of this region. Organotypic cultures can provide a cost-effective means to employ selective molecular biological strategies in elucidating cellular mechanisms of neuromodulation in the EC. We therefore adapted our acute slice model for organotypic culture applications and optimized a protocol for the preparation and biolistic transfection of cultured horizontal EC slices. Here, we present our detailed protocol for culturing EC slices. Using an *n*-methyl-d-glucamine (NMDG)-containing cutting solution, we obtain healthy EC slice cultures for electrophysiological recordings. We also present our protocol for the preparation of “bullets” carrying one or more constructs and demonstrate successful transfection of EC slices. We build upon previous methods and highlight specific aspects in our method that greatly improved the quality of our results. We validate our methods using immunohistochemical, imaging, and electrophysiological techniques. The novelty of this method is that it provides a description of culturing and transfection of EC neurons for specifically addressing their functionality. This method will enable researchers interested in entorhinal function to quickly adopt a similar slice culture transfection system for their own investigations.

## Graphical abstract

**Figure:**
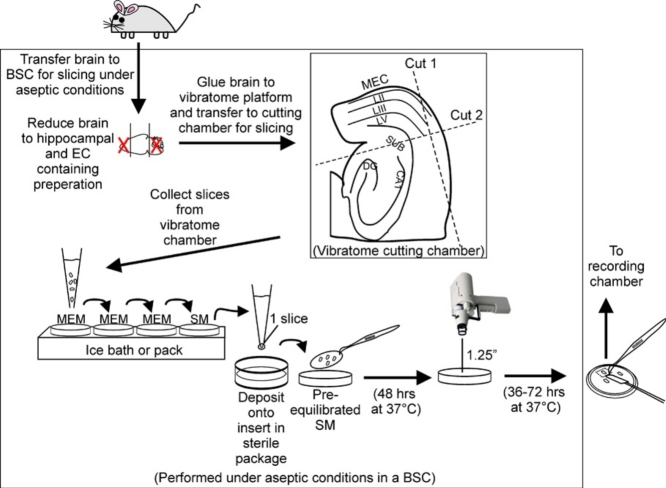

## Method details

### Animal Welfare Declaration

Animal procedures described here conformed to guidelines approved by the University of North Dakota Animal Care and Use Committee.

### Materials

**General resources**

• Chemical fume hood
• Isoflurane
• Bell jar
• Ice bucket
• 50 mL and 15 mL conical tubes
• Microcentrifuge tubes
• Biological safety cabinet (BSC)
• Vibratome (VT1000, Leica)
• Cyanoacrylate glue
• Millicell culture inserts (#picmorg50, Millicell)
• 40 × 11 mm petri dishes or 6-well plates
• 100 × 20 mm petri dish
• Ice packs
• 1 mL transfer pipette
• Sterile transfer pipette tips
• Sterile package of 18 in. × 26 in. surgical draping (4410-imc, IMCO products)
• 1 250 mL beaker
• 1 30 mL beaker
• 1 20 mL beaker
• 37 °C incubator
• Biorad Gene-Gun Low-Pressure System (#1652451, Biorad)
• Polyvinylpyrrolidone (PVP, #PVP360-100G, Sigma)
• Spermidine (#S2501-1G, Sigma)
• 1.6 μm gold microcarriers (#1652264, Biorad)
• Tefzel Tubing (#1652441, Biorad)
• Molecular biology grade ethanol
• Mini centrifuge (*e.g.* Daigger Sprout Mini-Centrifuge)
• Nitrogen tank
• Helium tank
• Serological pipettes
• Pipette aid
• Vacuum and syringe filters (0.22 μm)
• Autoclavable instrument bags

**Fig. 1:**
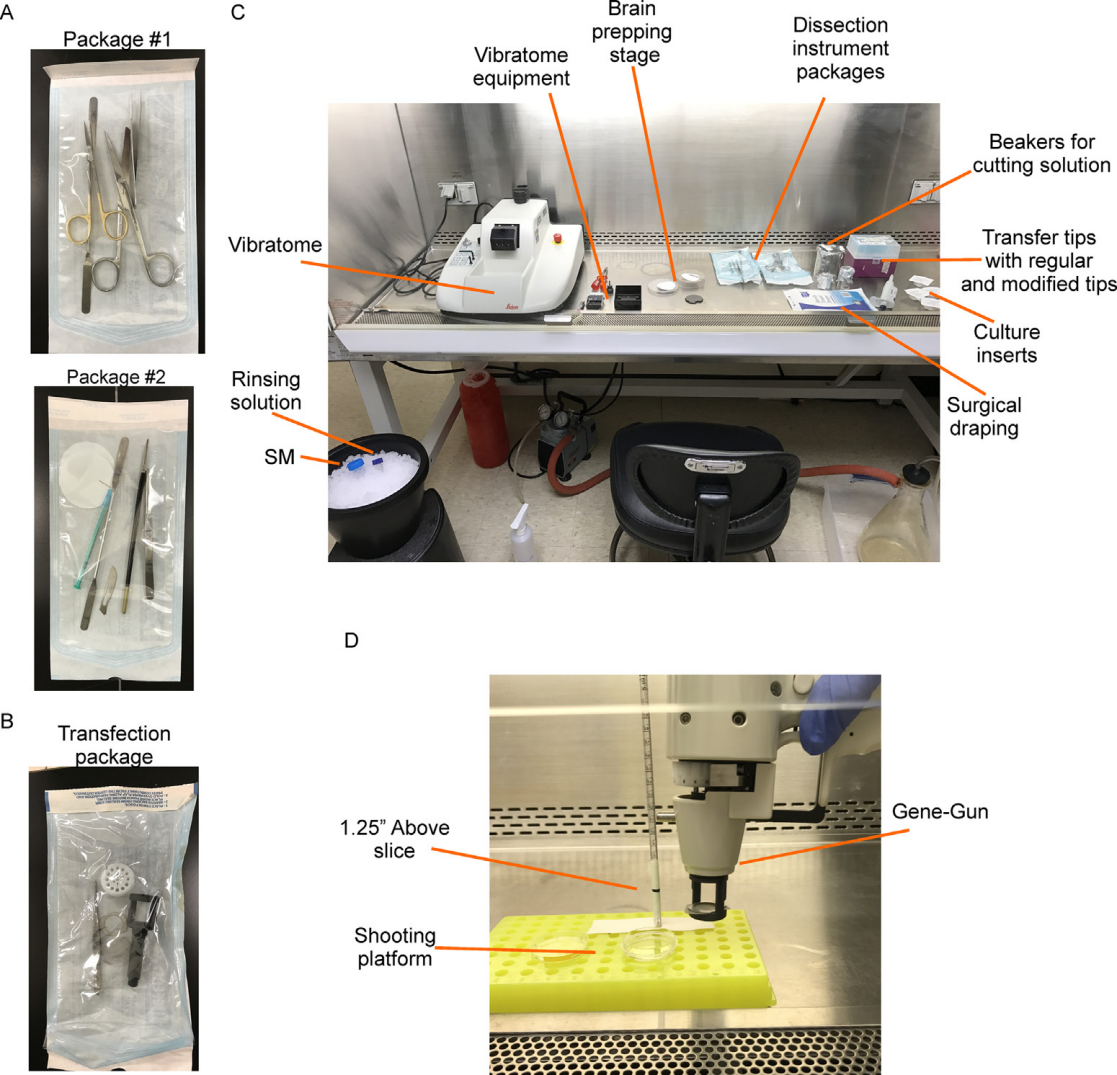
Equipment and setup for culturing and transfecting EC slices. A. *Top,* Surgical package #1 used for removal of the brain. *Bottom,* Package #2 used for further reducing the brain prior to and after slicing. B. Autoclaved tool package with contents necessary for one transfection condition. C. Basic layout of equipment in BSC prior to UV sterilization before the dissection and slicing procedure. The 4–40 mm petri dishes are absent from picture. D. Example of improvised transfection platform to improve the consistency of transfections.

**Supplies for dissection instrument packages** (Fig. 1A)

• Large Surgical scissors (#RS-6818, Roboz)
• Fine Surgical Scissors (#RS-5914, Roboz)
• 2 Fine forceps (#RS-5041, Roboz)
• 2 Spatulas (#13523, Ted Pella)
• 2 pieces of filter paper (#09-803-5A, Fisherbrand)
• 1 brush (11860, Ted Pella)
• 1 mL syringe with hand-bent 0.5 in. needle (we use this tool with the brush further dissect slices)
• Scalpel blade (RS-9861-21, Roboz)

**Supplies for transfection instrument packages** (Fig. 1B)

• Biorad Helios Gene-Gun barrel liner with rubber gasket
• Modified diffusing screen (#9317T109, McMaster-Carr) [1]
• Biorad diffuser screen washer (#1652475, Biorad)
• Fine forceps

### Recipes

1. **Cutting Solution (in mM)**: 130 *n*-methyl-d-glucamine-Cl (NMDG), 24 NaHCO_3_, 3.5 KCl, 1.25 NaH_2_PO_4_, 0.5 CaCl_2_, 5.0 MgCl_2_, and 10 glucose. Adjust pH to 7.3–7.4 and filter sterilize
2. **Rinsing Solution**: MEM (Life technologies cat #: 12360)
3. **Slice medium (SM)**: 50% HMEM without glutamine (Lonza cat#: 12-137F) + 25% heat-inactivated horse serum (Hyclone cat #: SH30074.03HI) + 25% HBSS (Life technologies cat #: 24020) + 2 mM glutamine (ThermoFisher cat#: 25030081), 5.95 mg/mL glucose, 100 ug/mL penicillin and streptomycin (100× Pen/strep stock; Cellgro cat#: 30-002-CI)
  a Adjust pH to 7.3 and osmolarity to ∼310 mOsm and filter sterilize

### Plasmid constructs

Plasmids carrying reporter genes for either green fluorescent protein (GFP, pGFP-V-RS) or red fluorescent protein (RFP, pRFP-C-RS) were purchased from OriGene (Rockville, MD). Plasmids were amplified in *E. coli* and isolated using a mega prep kit purchased from Qiagen (cat#: 12181). DNA working solutions used for cartridge preparation were diluted to 1 μg/μL in TE.

### Procedure overview

#### EC slice culture

**Setup for culturing procedure (estimated time∼1 h**)

1. Prepare and autoclave instrument packages and foil-covered 20, 30, and 250 mL beakers

a. Package #1: large surgical scissors, fine surgical scissors, fine forceps, spatula (Fig. 1A)

b. Package #2: spatula, scalpel blade, syringe with modified needle, brush, filter paper, fine forceps (Fig. 1A)

2. Aliquot necessary solutions in BSC and pre-equilibrate media

a. Aliquot ∼200 mL of filter sterilized cutting solution into a small sterile bottle for freezing at −80 °C

i. Critical step: Ensure that cutting solution is a very icy slurry prior to dissection to avoid poor slice health

b. Add ∼1.1 mL of SM to anticipated number of petri dishes/6-well plates needed

i. We typically culture 3–4 EC slices per insert and thus require between 4 and 6 inserts for one animal

ii. Pre-equilibrate dishes by placing them into a 37 °C humidified 5% CO_2_ incubator at least 30 min prior to dissection procedure

c. Aliquot ∼2 mL and ∼10 mL of SM and MEM, respectively, and place on ice for use during dissection procedure

3. Prepare and UV sterilize BSC for slicing (Fig. 1C)

a. Spray down BSC with 70% ethanol (EtOH) and lay out: vibratome equipment, both dissection packages, sterile beakers, 4–40 mm petri dishes, 1 mL transfer pipette, 1 mL transfer pipette tips, anticipated number of cell culture inserts, and cyanoacrylate glue

b. UV sterilize cabinet and materials for at least 15 min

4. Place foil covered sterile beakers on ice with SM and MEM

5. Use sterile scissors from dissection package to cut and modify tip of 1 mL transfer tip

a. These tips are used for transferring slice from cutting chamber, MEM washes, and placement on insert (see Graphical abstract)

6. Invert lids of 100 mm petri dish to use as brain-prepping stage (used prior to gluing brain to vibratome platform) and place filter paper atop

7. Fill vibratome trough with ice, turn on and prepare vibratome

8. Once cutting solution is sufficiently slushy and chilled, aliquot ∼150 mL into 250 mL beaker, ∼25 mL into 30 mL beaker, and ∼15 mL into 20 mL beaker in BSC

a. Recover with foil and return to ice bucket

**Dissection and slice collection procedure (estimated time ∼30 min)**

1. In a fume hood, deeply anesthetize animal in bell jar with isoflurane, spray animal with 70% EtOH, decapitate using large scissors from dissection package #1, and transfer head to 30 mL beaker of icy slurry solution

a. Replace foil, change gloves, and move to BSC with remaining tools in dissection package #1

2. On surgical draping, dissect brain into 20 mL beaker of icy solution and allow to cool for at least 1 min

a. Meanwhile, use EtOH to disinfect dissection region of BSC

3. Once the brain is chilled, pour off excess cutting solution and guide brain onto a piece of filter paper placed on prepping stage

4. With scalpel blade, gently invert brain so that it sits on its dorsal region and remove cerebellum and anterior portion of brain not containing hippocampus

5. Using the spatula and scalpel, carefully transfer the brain to the second piece of filter paper to remove excess cutting solution from the bottom (dorsal) part of the brain

6. Apply glue to vibratome platform, gently guide brain onto glue, transfer platform to cutting reservoir, and add ∼150 mL ice cold cutting solution without disturbing brain with large ice chunks

7. Collect 300 μm horizontal sections in reservoir

8. While slicing, remove an ice pack from the freezer and place in BSC; place the four petri dishes atop the pack

9. Using a paintbrush and modified syringe/needle, carefully isolate the EC by making two cuts (see Graphical abstract)

a. The first cut removes a portion of the perirhinal cortex and provides a visual cue for medial vs. lateral orientation at later stages in culture

b. The second cut near the hippocampal sulcus removes the hippocampus proper and leaves the EC intact

10. Fill three of the four dishes with chilled MEM and rinse slices by serial transfer, transferring as many slices as possible at once (see Graphical abstract)

11. Add cold SM to the fourth dish and transfer slices from MEM to SM

12. Remove pre-equilibrated dishes/plates from incubator and place in BSC

13. Open a new culture insert and transfer one slice at a time

a. Allow the slice to fall to the bottom of the modified tip prior to placement on insert (see Graphical abstract)

b. Once it is at the bottom, gently touch the solution bead containing the slice to the insert

c. Repeat for desired number of slices per insert

14. Switch to a regular 1 mL transfer tip and carefully remove excess SM around EC slices

a. Critical Step: Take care not to leave too much media on the slices

15. Using sterile forceps, transfer culture insert to pre-warmed media dish/plate

16. Repeat steps 13–15 until all slices have been successfully transferred

17. Clean and UV sterilize BSC

**Maintenance of EC slice cultures**

1. The media should be changed on day-in-vitro (DIV) 1 and then every two days, thereafter

2. On DIV 1, examine slices under an inverted light microscope and use a marker to denote the medial aspect of the slice on the bottom of the plate

#### Biolistic transfection of cultured EC slices

**Preparation of gene gun cartridges (estimated time ∼40 min)**

1. Insert a length of Tefzel Tubing into the tubing prep station and leave about a two-inch overhang at the end of the tubing rod

2. Turn on the N_2_ and open the tubing prep station regulator to just over 0.4 L per minute for about 30 min

3. Measure out 12.5–15 mg of 1.6 μm gold particles in a 1.5 mL microcentrifuge tube

4. Measure out 20 mg PVP into a 1.5 mL microcentrifuge tube and dissolve with 1 mL 100% EtOH

5. Aliquot 3.1 mL of 100% EtOH into a 15 mL tube and prepare a 50 μg/mL PVP dilution using the 20 mg/mL stock (7.75 μL of stock solution to 3.1 mL)

6. Prepare DNA working solution; we typically prepare 1 μg/μL dilutions of cDNA to be transfected

a. If more than one construct is to be transfected, we combine all constructs in a separate tube and mix by aspirating

b. Ratios will need to be determined for each condition and by individual investigators, but our results have indicated a high success rate of near equal co-transfection rates by simply using a 1:1 ratio of two constructs with the same promoter (see below)

7. To the gold, add 100 μL 0.5 M spermidine (filter sterilized); tap to mix and sonicate briefly

8. Add 50 μg of DNA to gold mixture; tap well to mix and sonicate very briefly

9. With the cap open, vortex on a low speed and add 100 μL of CaCl_2_ dropwise to the gold mixture

10. Sonicate briefly and tap well to mix

11. Allow 10 min for the mixture to precipitate while tapping every couple of minutes to re-agitate the mixture

12. Spin mixture for 30 s using a mini-centrifuge

13. Remove as much of the supernatant as possible without disturbing the gold pellet

14. Resuspend the mixture with 1 mL of 100% EtOH; tap mixture well and briefly sonicate to aid in resuspension

15. Repeat steps 12–14 for a total of three EtOH washes of gold pellets

16. Remove supernatant from final wash and resuspend pellet in 500 μL of the 50 μg/mL PVP solution

17. Tap this mixture very well to resuspend the pellet and transfer to a new 15 mL conical tube

18. Repeat this process (steps 16–17) in 500 μL iterations to thoroughly resuspend gold pellet

a. After transferring ∼1.5 mL, brief sonication may be employed to facilitate dissolving of remaining gold in PVP solution

19. Once 3 mL of the gold solution has been resuspended, turn off N_2_ and remove tubing from the prep station rod

20. Attach a piece of 18 in. length, 0.104 in. internal diameter silicone tubing to a 10 cc syringe and attach the other end of the silicone tubing to the Tefzel Tubing

21. Vigorously shake the suspension to resuspend and quickly draw up the solution into the Tefzel Tubing, leaving about 2 in. at the end of the tubing

22. Carefully, but quickly, place Tefzel Tubing into the prep station rod and mark where the solution stands in the tubing using a marker

23. With the syringe attached, wait five minutes to allow the gold to settle

24. After five minutes, draw off EtOH and rotate the tubing 180° and wait about 2 s before turning on constant rotation

25. Remove the syringe and continue to rotate for 30 s, after which turn on the N_2_ and continue rotating for another 5 min

26. Turn off N_2_, remove tubing, and use the Biorad tubing cutter to cut 0.5 in. cartridges from between the marked regions of the tubing

27. Store cartridges with a desiccant pellet in a 20 mL scintillation vial wrapped in parafilm at 4 °C

**Transfection procedure (estimated time ∼20 min)**

1. In a BSC, place the EtOH disinfected Biorad Helios Gene-Gun, autoclaved gene-gun transfection package (Fig. 1B), vials of gene gun cartridges, a sterile 40 mm petri dish, warmed SM, a 2 mL serological pipette, and a pipette-aid

a. We also include a shooting platform in the BSC to assist in consistent shooting distances (see below under Additional information)

b. A simple shooting platform can be constructed using a microcentrifuge tube rack that holds a rod upright (*e.g.* seriological pipette) marked at 1.25 in. distance from slice surface (Fig. 1D)

2. Open gene gun kits and load appropriate number of cartridges into cartridge holder using forceps

3. Connect gene gun to helium supply, as per manufacturer instructions, and adjust helium supply to 150 psi

4. Prime the gun by discharging twice with the cartridge holder positioned on an empty barrel

5. Remove EC slice dishes/plates from incubator and transfect one insert at a time

a. Transfer insert to a new empty sterile 40 mm petri dish and change the media on the dish/plate

b. Place dish containing only the insert on shooting platform, remove cover, position gun directly above insert at a distance of 1.25 in. away from slice surface and discharge cartridge

i. Critical Step: Slice health is important for obtaining quality electrophysiological recordings from slice cultures and we observed that shooting distance can drastically influence the quality of transfected, patchable cells

ii. Thus, using a shooting platform with a guide to improve consistency is strongly recommended (Fig. 1D)

c. Return insert to original dish containing fresh media

d. Repeat steps 5a–5c until all slices are transfected

i. Note: if transfecting with multiple constructs, be sure to use a different gene-gun instrument package with a clean mesh diffuser screen for each construct

6. Turn off helium source, return slices to incubator, clean up BSC, and return sealed cartridge constructs to 4 °C.

### Method validation

**Fig. 2:**
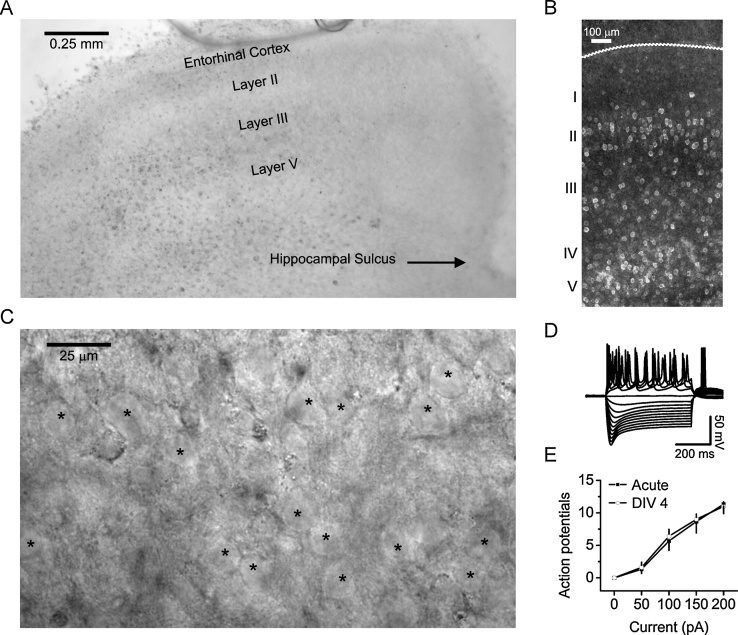
Healthy EC slices are prepared that exhibit typical cortical laminar distribution and the electrophysiological properties of principal neurons are generally well-preserved. A. DIC photograph of atypical EC slice section. B. NeuN immunofluorescent staining of organotypic cultures demonstrated that an abundant number of neurons were present and arranged in typical laminar fashion seen in acute slices. C. Higher magnification of a typical EC slice, imaged near layer II/layer III border, exhibited a respectable number of healthy and patchable principal neurons, indicated by asterisks (*). D. Example of voltage responses to a range of current injections obtained from a layer II principal neuron, confirming electrophysiological viability of neurons and typical signatures of this layer (*i.e.* preserved sag response). E. Input-output curve for number of action potentials generated per amount of direct-current injected, where responses from DIV 4 (from p12) slice cultures were compared with acute slices prepared from animals of the same litter (p16). No significant changes were observed (acute n = 3; DIV 4 n = 5).

We first ensured that cultured slices (Fig. 2A) maintained a laminar distribution and that we had healthy neurons in our preparation. Slices were fixed in 4% paraformaldehyde for 24 h, after which slices were individually transferred to frosted microscope slides (VWR #12-550-15). Immunostaining was performed using methods adapted from previous work [2]. Immunofluorescent staining for the neuronal marker NeuN (1:200, Milipore, #ABN78) demonstrated that EC layers were well-preserved in slice cultures and that neurons were detected after being in culture for several days (Fig. 2B). We next assessed slice health using electrophysiological methods described in previous work [2]. Under high-magnification, differential interference contrast microscopy (DIC) images revealed numerous healthy principal neurons for patching (Fig. 2C). Direct positive current injections to cultured layer II principal neurons (DIV 4) displayed action potential firing that was graded proportionally to current steps and negative current injections displayed early sag responses, typical of superficial layer II EC neurons. Input-output curves for action potential firing in response to current injections showed no significant difference between DIV 4 cultured slices and postnatal day 16 (p16) acute slices, suggesting no substantial changes in EC principal neuron excitability were present early on in culture.

**Fig. 3:**
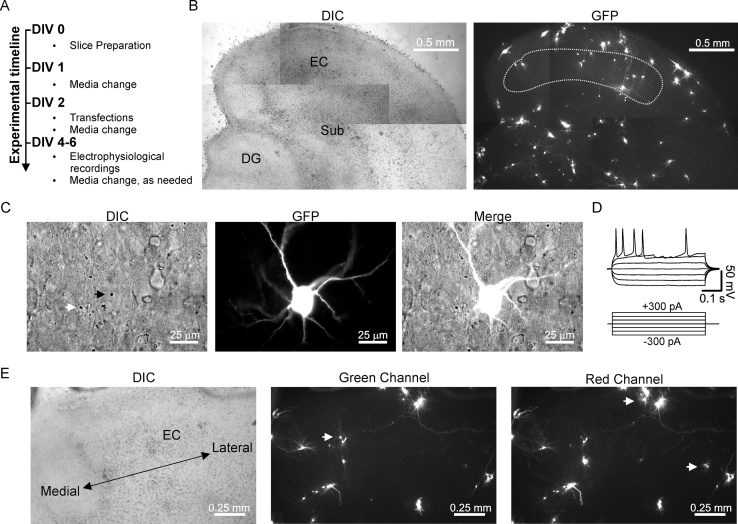
Optimized transfection scheme yields a reasonable number of healthy candidate neurons for whole-cell recordings in transfection experiments. A. Basic outline of slice culture preparation and transfection scheme. B. *Left,* DIC image for orientation of GFP-transfected EC slice. *Right,* green channel greyscale image of GFP transfected cells. Encircled region indicates many transfected cells in EC region of interest. C. Higher magnification of example GFP transfected neuron identified in layer III. *Left,* DIC image where black arrow denotes region containing transfected cell, indicated by the presence of a gold particle. Note, white arrow indicates gold particle where no cell was transfected. *Center,* green channel image of GFP-transfected neuron. *Right,* merge. D. Voltage responses to current injections taken from the neuron described in C. E. Example of a RFP and GFP co-transfected slice illustrating a high degree of co-transfection using a 1:1 ratio of plasmid DNA. This image was selected because it provides a few examples where some variable intensity existed, but the overall levels were largely similar between channels, indicated by white arrows. Arrows in either green channel (*middle*) or red channel (*right*) indicate relatively higher levels of expression for that cell in the respective channel.

Having validated that cultured EC slices were healthy and contained patchable neurons, we next wanted to validate our biolistic transfection procedures. Similar to other methods [3], we initially transfected slices within a few hours following the slicing procedure. Transfecting at two hours on DIV 0 resulted in several slices being lost from the insert following the pressure burst, likely owing to insufficient time for adherence to the membrane. Furthermore, we observed consistently higher transfection rates when slices were permitted to acclimate to culture conditions for a few days. The additional time in culture likely allowed the slice to reach a new physiological steady-state and permitted intrinsic processes to clear away unhealthy cells and other debris, which may have otherwise obstructed the penetrating transfection particles from reaching healthy neurons. For these reasons, we adopted a transfection scheme where slices were transfected on DIV 2 and experiments were conducted 36–72 h later (Fig. 3A). It is important to point out that because the culturing procedure results in massive denervation of the EC and because denervation can result in the occurrence of homeostatic changes in the target cell [4], careful attention should be given to time points of culturing, transfecting, and recording to ensure consistency across experiments. This method provided high transfection rates per slice in EC regions of interest (Fig. 3B). Recordings from layer III EC neurons transfected with GFP remained healthy and continued to be electrically active (Fig. 3C and D). Because some experimental conditions may require co-transfection of multiple plasmids to permit either expression of reporter molecules or because conditions may require introduction of multiple protein components, we assessed transfection ratios of 1:1 plasmid DNA constructs (*i.e.* 25 μg of each plasmid added during cartridge preparation) using GFP and RFP reporters to evaluate levels of co-expression. Under these conditions, we consistently observed a prominent level of co-expression across many experiments spanning different slice culture batches, although minor variations in intensity were occasionally seen in a few cells (Fig. 3E, *see white arrows*). These results suggest that a 1:1 ratio may be sufficient when introducing multiple gene products. However, for experiments where one of the constructs is simply a reporter (*e.g.* GFP), we adjust our ratios to 0.75:0.25 for gene of interest to reporter. Taken together, our results demonstrate that this method provides several candidate cells for patching following transfection and that transfected neurons remain healthy.

### Troubleshooting

The following is a list of potential problems and suggestions to improve outcomes based on our experiences:

1. Problem: Slices dry out and die

a. Potential Solutions:

i. The best way to reduce this problem was by reducing time spent with the petri dish cover off in BSC while placing slices on inserts

ii. Also, a breathable humidity chamber within the incubator was also useful as high humidity levels were crucial to slice survival

2. Problem: DNA-gold mixture is not dissolving

a. Potential Solutions:

i. The mixture is tough to dissolve but this process is aided by constant brief sonication

ii. If it is impossible to dissolve, double-check the DNA stock solution concentration to ensure its accuracy; if more than 50 μg is added, it is nearly impossible to dissolve the clump, in which case it is best to start over

3. Problem: Transfected slices are uneven, in that some slices are transfected while others are blank

a. Potential Solutions:

i. This problem is likely due to poor quality bullets and is likely the result of poor spreading of gold mixture during the coating stage; there should not be a visible gold line in the cartridge

ii. Better bullets can be generated by ensuring a well dissolved mixture and spinning the tubing for a longer duration after drawing off the EtOH, but before turning on the N_2_

4. Problem: Both transfected and non-transfected cells from a shot culture dish are visible but very difficult to patch and seem to be encased in some difficult-to-penetrate matrix

a. Potential Solutions:

i. Use a control non-transfected (*i.e.* non-shot) plate to ensure cell viability; if cells are visible and patchable, the problem is almost certainly due to pressure intensity or distance of shot from the top of the slice

ii. We strongly recommend using at least 1.25 in. shooting distance from the top of the slice to the barrel liner

## Additional information

### Background and discussion

Organotypic cultures are a well-established *in vitro* model to investigate the cytoarchitecture and neurophysiology of brain tissue [5], [6]. Whereas such cultures comprised of hippocampal regions have been utilized by many labs, relatively fewer reports involve organotypic cultures consisting of entorhinal tissue. Horizontal organotypic slices consisting of both entorhinal and hippocampal regions of P0–P5 animals have been employed to study axonal regeneration and synaptic scaling at the dentate gyrus following lesions to the perforant pathway *in vitro* [4], [7]. Our method is unique in that it specifically details the culturing and transfection protocols used for the electrophysiological investigations of EC neurons and will be helpful for mechanism studies involving EC neuromodulation.

There are several steps we employ that distinguish our method from other methods of organotypic slice culture. First, we have chosen to use a vibratome as opposed to either a tissue chopper or slicer that others have described [6], [8], [9]. Although use of a vibratome requires additional time for collecting tissue, we find that it produces healthy slices that are well-suited for electrophysiological recordings as previously demonstrated by others, *e.g.* in spinal cord organotypic cultures [10]. Second, we choose to utilize a horizontal preparation because this approach provides slices that are similar in orientation to our acute model. Third, unlike most organotypic slice culture methods, we use a cutting solution where Na^+^ has been largely replaced with NMDG, in order to minimize excitotoxicity that may occur during the traumatic slicing process. Alternative methods to minimize excitotoxicity involve supplementing the cutting solution with antioxidants, such as pyruvate and ascorbic acid [8], [11]. NMDG-containing cutting solutions have become widely adopted in electrophysiological laboratories working with acute slices because these steps are neuroprotective, especially toward the more vulnerable interneuron populations [12]. Overall, our EC slice model attempts to yield a similar quality of initial tissue preparation for longer-term culture that resembles our acute slice model and we have had success culturing slices using animal ages ranging from p6 to P18.

Our optimized transfection conditions are informed by both the instruction manual provided with the Biorad Helios Gene-Gun, as well as other detailed published protocols describing biolistic cartridge preparation and transfection [1], [9], [13]. The reader is referred to those references for additional detail, however, we obtained excellent results in our method by employing a few modifications. First, it is essential that the gold mixture be homogenously distributed about the cartridge to increase transfection efficiency for all available slices on a plate. We found that including a more thorough sonication periods of the DNA gold mixture was necessary to obtain a better suspension for coating the Tefzel Tubing. Additionally, a longer period of tubing rotation following EtOH removal, but prior to turning on N_2,_ achieved an improved mixture distribution. Secondly, neurons are very susceptible to the pressure burst during the transfection procedure and the quality of cells available for electrophysiological recordings are greatly influenced by the shooting distance used during the transfection. Initially, we held the gun and barrel liner at a distance similar to what other methods reported. On recording days, we observed an unknown matrix that seemed to encase the neurons and impede our ability to form tight gigaohm seals with transfected neurons. However, when the shooting distance was increased, this matrix was no longer a problem and whole-cell success rates were substantially improved. We therefore addressed this problem by optimizing a shooting a distance for our 11 mm petri dishes, which we empirically found to be at 1.25 in. or greater. To improve consistency across experiments, we improvised a shooting platform that was clearly demarcated with the ideal shooting distance for an easy reference (see Fig. 1D). Thirdly, although not rigorously tested, we also found that the media composition seemed to influence the quality of transfected cell-health for recordings. Initially, we were able to obtain healthy slice cultures using a more simplistic media recipe described by other labs [3]. However, transfection efficiency seemed to be less than ideal when following the biolistic transfection process. We observed a consistent improvement in efficiency after modifying the SM with the addition of glucose and glutamine, similar to the Stoppini method [6] (see under *Recipes* above). Finally, we report that transfecting with GFP and RFP constructs in a 1:1 ratio provided a high degree of co-transfection, in agreement with a recent report involving biolistic double-transfections [8]. Ideally, co-transfection experiments should use plasmids containing the same promoter, as was the case in our experiment. Results are expected to vary if different and/or less efficient promotor-carrying plasmids are to be used, in which case ratios will need to be determined by individual investigators.

We expect this method to be useful for other investigators interested in addressing molecular and cellular mechanisms of entorhinal neurophysiology. The details described herein will assist others in quickly adopting a similar EC slice culture method for their lab. Additionally, this information may be useful to those troubleshooting biolistic transfection methods in other brain regions.
